# *CMTR1* is associated with increased asthma exacerbations in patients taking inhaled corticosteroids

**DOI:** 10.1002/iid3.73

**Published:** 2015-07-14

**Authors:** Amber Dahlin, Joshua Denny, Dan M. Roden, Murray H. Brilliant, Christie Ingram, Terrie E. Kitchner, James G. Linneman, Christian M. Shaffer, Peter Weeke, Hua Xu, Michiaki Kubo, Mayumi Tamari, George L. Clemmer, John Ziniti, Michael J. McGeachie, Kelan G. Tantisira, Scott T. Weiss, Ann Chen Wu

**Affiliations:** ^1^ Channing Division of Network Medicine, Department of MedicineBrigham and Women's Hospital and Harvard Medical SchoolBostonMassachusetts02115; ^2^ Department of Medical BioinformaticsVanderbilt University School of MedicineNashvilleTennessee37235; ^3^ Department of MedicineVanderbilt UniversityNashvilleTennessee37235; ^4^ Division of Clinical Pharmacology, Department of MedicineVanderbilt University School of MedicineNashvilleTennessee37235; ^5^ Center for Human GeneticsMarshfield Clinic Research FoundationMarshfieldWisconsin54449; ^6^ Biomedical Informatics Research CenterMarshfield Clinic Research FoundationMarshfieldWisconsin54449; ^7^ Department of CardiologyCopenhagen, University HospitalGentofteDenmark; ^8^ School of Biomedical InformaticsThe University of Texas Health Science Center at HoustonHoustonTexas77030; ^9^ Riken Center for Genomic MedicineKanagawaJapan; ^10^ Center for Child Health Care Studies, Department of Population MedicineHarvard Pilgrim Health Care Institute and Harvard Medical SchoolBostonMassachusetts02115

**Keywords:** Asthma, GWAS, inhaled corticosteroids, EMR, exacerbations, pharmacogenomics

## Abstract

Inhaled corticosteroids (ICS) are the most effective controller medications for asthma, and variability in ICS response is associated with genetic variation. Despite ICS treatment, some patients with poor asthma control experience severe asthma exacerbations, defined as a hospitalization or emergency room visit. We hypothesized that some individuals may be at increased risk of asthma exacerbations, despite ICS use, due to genetic factors. A GWAS of 237,726 common, independent markers was conducted in 806 Caucasian asthmatic patients from two population‐based biobanks: BioVU, at Vanderbilt University Medical Center (VUMC) in Tennessee (369 patients), and Personalized Medicine Research Project (PMRP) at the Marshfield Clinic in Wisconsin (437 patients). Using a case–control study design, the association of each SNP locus with the outcome of asthma exacerbations (defined as asthma‐related emergency department visits or hospitalizations concurrent with oral corticosteroid use), was evaluated for each population by logistic regression analysis, adjusting for age, gender and the first four principal components. A meta‐analysis of the results was conducted. Validation of expression of selected candidate genes was determined by evaluating an independent microarray expression data set. Our study identified six novel SNPs associated with differential risk of asthma exacerbations (*P* < 10^−05^). The top GWAS result, rs2395672 in *CMTR1*, was associated with an increased risk of exacerbations in both populations (OR = 1.07, 95% CI 1.03–1.11; joint *P* = 2.3 × 10^−06^). Two SNPs (rs2395672 and rs279728) were associated with increased risk of exacerbations, while the remaining four SNPs (rs4271056, rs6467778, rs2691529, and rs9303988) were associated with decreased risk. Three SNPs (rs2395672, rs6467778, and rs2691529) were present in three genes: *CMTR1*, *TRIM24* and *MAGI2*. The *CMTR1* mRNA transcript was significantly differentially expressed in nasal lavage samples from asthmatics during acute exacerbations, suggesting potential involvement of this gene in the development of this phenotype. We show that genetic variability may contribute to asthma exacerbations in patients taking ICS. Furthermore, our studies implicate *CMTR1* as a novel candidate gene with potential roles in the pathogenesis of asthma exacerbations.

## Introduction

Asthma, a complex disease characterized by airway hyper‐responsiveness, airway obstruction, inflammation and airway remodeling, confers substantial global financial burden and is a significant source of increasing morbidity and mortality worldwide 1, 2. In the majority of cases, asthma can be managed in an outpatient setting. However, some severely affected asthmatics, including patients with poorly controlled asthma, experience exacerbations, defined as the occurrence of hospitalizations, emergency department (ED) or intensive care unit (ICU) visit to receive treatment, and the requirement of oral corticosteroids. The frequency of exacerbations and/or need for oral corticosteroids can be used as a measure of asthma severity 3.

Clinical treatment of asthma exacerbations consumes significant healthcare resources, and patients who experience severe exacerbations have more ED and ICU visits, more frequent and longer hospitalizations related to their asthma, increased numbers of lost school and work days, more frequent unscheduled physician visits, diminished quality of life, and increased mortality risk 3, 4. Inhaled corticosteroids (ICS), which are the most commonly prescribed asthma controller medications, reduce airway inflammation and improve asthma symptoms, thereby diminishing the likelihood of exacerbations. While most patients' symptoms are well controlled with regular ICS use, with or without concomitant use of long‐acting *β*_2_‐agonists (LABAs), up to 30% of individuals who adhere to controller therapy still respond minimally or not at all, and experience persistent symptoms that can lead to the development of exacerbations 5. An increasing number of studies reveal that genetic variation due to single nucleotide polymorphisms (SNPs) in various genes substantially contributes to the heterogeneity in clinical responsiveness to ICS 6, 7, 8, 9, 10, 11, 12, 13, 14, 15, 16, 17, 18, 19, 20, 21, 22, 23. Furthermore, recent GWAS and candidate gene studies have identified genetic risk factors for asthma exacerbations, specifically the candidate genes *ST13* 24, *ADRB2* 10, 14, 25, *P2X7* 26, *FCER2* 21, 27, and *IL13* 28, in patients taking ICS.

Although promising, these results do not entirely explain the heterogeneity of asthma severity despite ICS treatment, indicating that additional genetic and environmental risk factors are involved. In light of these findings, we have hypothesized that novel genetic loci contribute to the development of asthma exacerbations. In this study, we performed a genome‐wide screen of two independent asthmatic populations taking ICS, in an effort to identify pharmacogenetic predictors for asthma exacerbations.

## Methods

### Populations and measures

Demographic information for the populations investigated in this study is provided in Table 1. Samples and clinical data were obtained from two population‐based biobanks: BioVU 29 at Vanderbilt University Medical Center (VUMC) in Tennessee, which served as the discovery population, and the Personalized Medicine Research Project (PMRP) 30 at the Marshfield Clinic in Wisconsin, which served as the replication population. As previously described, BioVU is the nation's largest biobank, as it contains DNA linked to the de‐identified electronic medical records (EMR) of >160,000 unique individuals, of which ∼80% are of European descent 29, 30, 31. Similarly, the PMRP is comprised of 20,000 non‐Hispanic whites (98%) from Central Wisconsin 30. Enrollment was conducted in accordance with the guidelines established by the Institutional Review Boards of the VUMC and Marshfield Clinic Research Foundation. Access and evaluation of both BioVU and PMRP was obtained through the Pharmacogenomics Discovery and Replication in Large Populations (PGPop) Consortium (http://pgpop.mc.vanderbilt.edu/labkey/project/PGPop/begin.view).

**Table 1:** Demographic information for BioVU and PMRP

Characteristic	Exacerbations, no. [%]	No exacerbations, no. [%]
BioVU, total	64 [17.3]	305 [82.7]
Age, mean (range), years	17.5 (3–35)	23.4 (4–42)
Sex		
Male	42 [65.6]	215 [70.5]
Female	22 [34.4]	90 [29.5]
PMRP, total	207 [47.4]	230 [52.6]
Age, mean (range), years	31.2 (18–66.5)	32.1 (18–64.1)
Sex		
Male	53 [25.6]	71 [30.9]
Female	154 [74.4]	159 [69.1]

Age represents the age of the patients at the time of enrollment.

### Subject selection and phenotyping

Both study populations were identified with an electronic algorithm (available at http://PheKB.org) that was rigorously developed and validated at each site by chart review of 50 randomly selected patients. Using this algorithm, we selected Caucasian patients with a principal ICD‐9 diagnosis of asthma (493.xx), who had at least two clinical encounters related to their asthma on two different days, and who had a prescription for an ICS (beclomethasone, budesonide, ciclesonide, flunisolide, mometasone, or triamcinolone). Patients had initiated ICS treatment prior to the exacerbation event. We excluded patients with confounding diseases such as cystic fibrosis, chronic obstructive pulmonary disease, pulmonary hypertension, pulmonary embolism, lung cancer, immunodeficiency, bronchiectasis, congestive heart failure, and other diseases. We also obtained patient demographic information, including age at enrollment and gender, for use as covariates in regression models.

The primary outcome measure was asthma exacerbations, where we defined an “exacerbation” as an asthma‐related hospitalization or ED visit that included evidence of oral or intravenous corticosteroid use during the stay, and an ICD‐9 code of asthma (493.xx) or wheezing (786.07) during the admission. If a patient had both a hospitalization and ED visit during the same time period, we only counted the hospitalization. We specified exacerbations as a binary variable (coded 0 or 1), where a value of 1 was coded if there was a record of at least one reported hospitalization or ED visit related to asthma over the year preceding the study visit for sample collection, while a value of 0 corresponded to a null report of exacerbations.

### Genotyping and data quality control

Whole blood samples were obtained from patients at each study site, and genomic DNA was extracted from the buffy‐coat fraction of the blood samples using the QIAamp DNA blood mini kit (Qiagen, Germantown, MD) according to the manufacturer's protocol. Exome array genotyping and analysis of array data was performed using Illumina's Omni2.5 Exome BeadChip (Illumina, Inc., San Diego, CA) system at the Riken Center for Genomic Medicine (Japan). The samples from BioVU and PMRP were analyzed separately from one another. The software packages PLINK v.1.07 32 and GenABEL 33 were used for QC of genotype data. The exome array datasets from BioVU (731,390 SNPs) and PMRP (662,256 SNPs) were first merged and pruned to obtain 740,924 common autosomal SNPs. These SNPs were then filtered to exclude SNPs with >10% missing genotypes (over all patients; 93,029 SNPs), SNPs with low MAF (<5%) (134,059 SNPs), and SNPs with Hardy–Weinberg equilibrium *P* values among controls below 10^−04^ (64 SNPs). Five patients were excluded from BioVU for high autosomal heterozygosity (FDR <1%). The average genotyping completion rate for each patient was 99% for BioVU and 100% for PMRP. Prior to running the PCA‐adjusted GWAS, non‐independent markers, that is, those in approximate linkage equilibrium based on pairwise genotypic correlation, were pruned out using a window of 50 SNPs, a step size of 5 SNPs and *r* ^2^ threshold of 0.5, resulting in a final dataset that included 806 patients (369 patients from BioVU and 437 patients from PMRP) and 237,726 common, independent SNPs.

### Statistical methods

The R software package GenABEL 33 was used for variant‐level logistic regression testing with principal components analysis (PCA) and covariate adjustment. We evaluated BioVU as the discovery population and PMRP as the replication population due to the larger size of PMRP. Initial GWA analyses for both populations were performed using the same procedures. The phenotype (frequency of exacerbations) was evaluated as a binary outcome variable coded 0 or 1, where 0 indicates no report of exacerbations and 1 indicates the occurrence of one or more reported exacerbations during the two year study period. The effect sizes (odds ratios with 95% confidence intervals) for each marker were calculated using logistic regression, assuming an additive genetic model. Population stratification was corrected by PCA adjustment, including the first four principal components as covariates, in addition to age and gender, in the logistic regression model. Significance of association with exacerbations was assessed for each marker using the *χ* ^2^ test, and a Bonferroni‐corrected genome‐wide significance threshold for all markers was set at 2.1 × 10^−07^ (for 237,726 tests). A suggestive genome‐wide P value threshold of 1 × 10^−05^ was applied for screening of markers in the discovery stage, while a nominal *P* value threshold of 0.05 in the replication population was used to validate SNPs from the discovery set. A meta‐analysis of fixed effects was performed on SNPs common to both populations, and *P* values from the two GWA studies were combined using a weighted Z test 34. In the meta‐analysis, SNPs approaching genome‐wide significance in BioVU (*P* < 10^−05^), with one‐sided *P* values <0.05 in PMRP and meeting a joint *P* < 0.05 threshold, while also possessing concordant directions of effect in both populations, were considered significant.

### Analysis of mRNA expression data

The expression of mRNA in nasal lavage samples from children with asthma during exacerbations, and 7–14 days following exacerbation, has been described by Bosco, et al.^35^. We downloaded the normalized mRNA expression data corresponding to this study from the Gene Expression Omnibus (GEO; GEO Accession GSE30326) to evaluate expression levels of transcripts potentially involved in asthma exacerbations. A differential expression analysis of data from 32 paired samples during and post‐acute exacerbation was performed using the Bioconductor package *limma* 36. Probe sets corresponding to the mRNA transcripts for the genes associated with exacerbations listed in Table 3 were evaluated for differential expression. Global significance was first determined using *limma*, and the significance of any differentially expressed mRNA was then assessed for the paired samples (post‐exacerbation vs. during exacerbation) using Welch's two‐sample *t* test, implemented in Bioconductor.

## Results

The study population (Table 1) included a total of 806 Caucasian patients (369 from BioVU [Vanderbilt]) and 437 from PMRP [Marshfield]). The mean age was 17.5 years in BioVU and 31.2 years in PMRP (Table 1). Over all, in BioVU, 17% of patients experienced severe asthma exacerbations compared to 47% in PMRP (Table 1). The increase in exacerbations in PMRP relative to BioVU may be due to the larger number of adults in PMRP, who experience more frequent hospitalizations due to asthma 37. In addition, this population included a larger number of females, who exhibit more frequent exacerbations in adulthood 38. In both populations, a minority of patients experienced only one exacerbation (32% in BioVU and 44% in PMRP) while the majority experienced ≥2 exacerbations (68% in BioVU and 56% in PMRP) during the study period.

**Figure 1:**
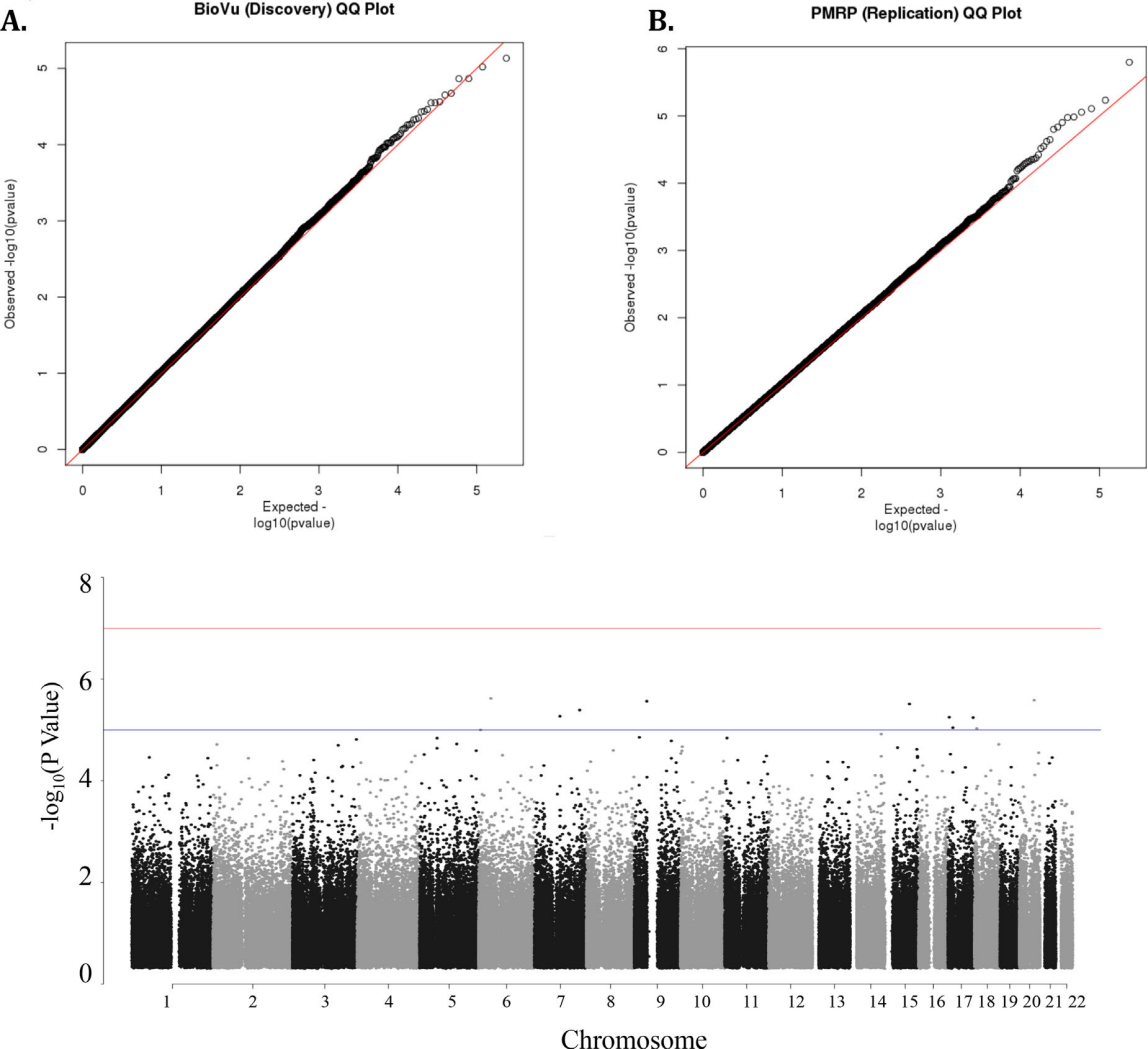
**GWAS results.** Plots of –log_10_ transformed, two‐sided *P* values (expected vs. observed) for (A) BioVU and (B) PMRP are shown (*λ* [SE]: in [A] 1.03 [5.4 × 10^−05^]; in [B] 1.01 [7.6 × 10^−06^]). (C) The Manhattan plot shows –log_10_‐transformed association *P* values for the meta‐analysis, plotted versus chromosome position (*x* axis). The results were adjusted for age at reported exacerbation event, gender, and the first four principal components. The blue line indicates the *P* value threshold for suggestive genome‐wide significance (10^−05^) whereas the red line indicates the genome‐wide significance threshold (10^−07^).

We first performed individual GWA analyses in each population, specifying BioVU as the discovery population, and then conducted a meta‐analysis of the results. The corresponding quantile–quantile (Q–Q) plot of the discovery analysis (Fig. 1A) demonstrates that the SNPs with the lowest *P* values deviate from what is expected for a null distribution, suggesting that some may reflect true pharmacogenetic associations with asthma exacerbations in ICS users. The genomic inflation factor *λ* was 1.03, indicating that minimal population stratification is present. The Manhattan plot (Fig. 1C) shows that the regions of SNPs that are most significantly associated with exacerbations are in chromosomes 6, 7, 9, 15, 17, 18, and 20. While none of the discovery GWAS SNPs surpassed the threshold for genome‐wide significance, four SNPs had *P* values suggestive of genome‐wide significance (the top 25 SNPs are listed, Table 2). Thirteen SNPs were found within the intronic regions of 13 genes, with the highest ranked SNP present in *protein tyrosine phosphatase, receptor type, T* (*PTPRT*; Table 2). Among the top discovery SNPs, rs2395672 in *cap methyltransferase 1* (*CMTR1*) was significantly associated with increased risk of exacerbations in both BioVU and PMRP (Tables 2 and 3).

**Table 2:** Top discovery GWAS SNP associations (*P* < 10^−05^)

SNP	Chr.	A1	A2	Gene symbol	Effect	OR (95%CI)	*X* ^2^	*P* Value
rs8119002	20	T	C	*PTPRT*	Silent	1.07 (1.04–1.10)	20.1	3.67 × 10^−06^
rs7900146	10	A	G			1.15 (1.08–1.22)	19.6	4.78 × 10^−06^
rs6070784	20	G	A			1.08 (1.04–1.12)	18.9	6.79 × 10^−06^
rs11215855	11	C	T			0.97 (0.96–0.98)	18.9	6.81 × 10^−06^
rs6026344	20	G	A	*APCDD1L‐AS1*	Silent	1.03 (1.02–1.04)	18.1	1.06 × 10^−05^
rs235324	21	T	C	*PTTG1IP*	Silent	1.13 (1.07–1.19)	18.0	1.12 × 10^−05^
rs4800012	18	G	A			1.15 (1.08–1.22)	17.6	1.37 × 10^−05^
rs12415740	10	G	A			1.04 (1.02–1.05)	17.5	1.41 × 10^−05^
rs12359379	10	A	G	*KCNMA1*	Silent	1.05 (1.03–1.08)	17.5	1.41 × 10^−05^
rs12264884	10	A	G			1.04 (1.02–1.06)	17.2	1.72 × 10^−05^
rs13421149	2	T	C			1.06 (1.03–1.09)	17.1	1.82 × 10^−05^
rs2395672	6	G	A	*CMTR1*	Silent	1.08 (1.04–1.12)	17.0	1.86 × 10^−05^
rs2283524	16	G	A	*HS3ST2*	Silent	1.04 (1.02–1.06)	16.7	2.24 × 10^−05^
rs2430893	18	C	T	*WDR7*	Silent	1.16 (1.08–1.24)	16.6	2.31 × 10^−05^
rs10503031	18	A	C	*CCBE1*	Silent	1.04 (1.02–1.06)	16.6	2.36 × 10^−05^
rs1402012	3	G	A			1.14 (1.07–1.21)	16.3	2.64 × 10^−05^
rs9354178	6	T	C	*EYS*	Silent	0.96 (0.95–0.98)	16.3	2.75 × 10^−05^
rs480478	11	C	T			1.08 (1.04–1.12)	16.3	2.76 × 10^−05^
rs13329064	14	T	C			1.12 (1.06–1.18)	16.1	3.03 × 10^−05^
rs4671340	2	T	C			1.07 (1.03–1.10)	16.1	3.08 × 10^−05^
rs11652685	17	A	G	*BZRAP1*	Silent	1.12 (1.06–1.18)	16.0	3.19 × 10^−05^
rs17098161	12	T	C	*PPM1H*	Silent	1.13 (1.06–1.20)	15.8	3.58 × 10^−05^
rs2421748	3	T	G	*MECOM*	Silent	1.15 (1.07–1.23)	15.7	3.77 × 10^−05^
rs7251403	19	T	C	*CACNA1A*	Silent	1.07 (1.04–1.11)	15.6	3.95 × 10^−05^
rs2220885	11	T	C			1.06 (1.03–1.09)	15.6	3.99 × 10^−05^

Outcome is asthma‐related hospitalizations or ED visits while taking inhaled corticosteroids. SNP, single nucleotide polymorphism; Chr., chromosome; A1, reference allele; A2, variant allele; Gene symbol, NCBI gene symbol; Effect, predicted SNP effect on amino acid sequence; OR, odds ratio for the homozygous variant genotype (additive genetic model); 95%CI, 95% confidence interval for the Odds ratio; *χ* ^2^, *χ* ^2^ statistic; *P* value, one‐sided association *P* value. All SNPs have MAF >5%.

In the meta‐analysis, while none of the SNPs achieved genome‐wide significance, the joint *P* values for six SNPs sharing the same direction of effect in both populations were suggestive of genome‐wide significance (listed in Table 3). Three SNPs were present within three genes, of which the “AA” variant genotype for the top association, rs2395672 in *CMTR1*, had the greatest effect (pooled OR 1.07 [CI = 1.03‐1.11]; Table 3). The top two SNPs, rs2395672 and rs279728, were associated with increased exacerbations risk in both populations, while the remainder was associated with reduced risk (Table 3).

**Table 3:** Replicated SNP associations

						BioVU		PMRP		Meta‐analysis	
SNP	Chr.	A1	A2	Gene symbol	Effect	OR (95%CI)	*P* value	OR (95%CI)	*P* value	OR (95%CI)	Joint *P* value
rs2395672*	6	G	A	*CMTR1*	Silent	1.08 (1.04–1.12)	1.86 × 10^−05^	1.05 (1.01–1.10)	6.00 × 10^−03^	1.07 (1.03–1.11)	2.32 × 10^−06^
rs279728	20	T	G			1.02 (1.00–1.04)	6.45 × 10^−03^	1.04 (1.02–1.06)	5.67 × 10^−05^	1.03 (1.01–1.05)	2.64 × 10^−06^
rs4271056	9	C	T			0.96 (0.92–0.99)	6.71 × 10^−03^	0.92 (0.88–0.96)	5.69 × 10^−05^	0.94 (0.90–0.97)	2.77 × 10^−06^
rs6467778	7	T	C	*TRIM24*	Silent	0.99 (0.97–1.00)	2.14 × 10^−02^	0.96 (0.95–0.98)	1.89 × 10^−05^	0.97 (0.96–0.99)	4.18 × 10^−06^
rs2691529	7	A	C	*MAGI2*	Silent	0.97 (0.93–1.01)	5.08 × 10^−02^	0.90 (0.86–0.94)	6.26 × 10^−06^	0.93 (0.89–0.97)	5.57 × 10^−06^
rs9303988	18	T	C			0.97 (0.95–1.00)	1.20 × 10^−02^	0.95 (0.92–0.97)	1.12 × 10^−04^	0.96 (0.93–0.99)	9.52 × 10^−06^

SNP, single nucleotide polymorphism; Chr., chromosome; A1, reference allele; A2, variant allele; Gene symbol, NCBI gene symbol; Effect, predicted SNP effect on amino acid sequence; OR, odds ratio for the homozygous variant genotype (additive genetic model); 95%CI, 95% confidence interval for the Odds ratio; Joint *P* value, Liptak‐combined *P* values. All SNPs have MAF >5%. *SNP was represented among the top discovery GWAS results (listed in Table 2).

**Figure 2:**
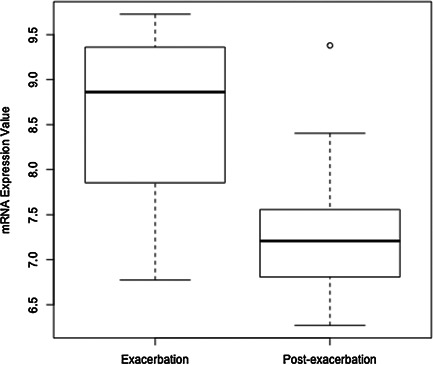
**Differential Expression of *CMTR1*.** The expression of *CMTR1* mRNA transcripts was evaluated using a publicly available dataset (GEO accession GSE30326) from a microarray analysis of nasal lavage samples obtained from asthmatic children during and after asthma exacerbation 35. The *y* axis represents normalized expression values of *CMTR1* from 32 paired mRNA samples.

To further investigate whether any of the genes listed in Table 3 (*CMTR1, tripartite motif containing 24* [*TRIM24*], and *membrane associated guanylate kinase, WW and PDZ domain containing 2* [*MAGI2*]) were potentially involved in the pathogenesis of asthma exacerbations, we examined the relative expression levels of these genes in patients experiencing exacerbations 35. Of these genes, *CMTR1* alone was significantly differentially expressed in paired nasal lavage samples obtained from asthmatic children 1–2 weeks following an acute exacerbation versus during the exacerbation event (Fig. 2). Following exacerbation, but not during exacerbation, *CMTR1* expression was significantly reduced (log_FC_ = −1.29, adjusted *P* value = 0.016, unadjusted *P* value = 1.1 × 10^−04^). Neither *TRIM24* (log_FC_ = −0.21, adjusted *P* value = 0.55, unadjusted *P* value = 0.42) nor *MAGI2* (log_FC_= 0.187, adjusted *P* value = 0.47, unadjusted *P* value = 0.34) demonstrated significant changes in mRNA expression by exacerbation status.

## Discussion

The main finding of this genetic association study is that *CMTR1* is associated with an increased risk of asthma exacerbations. Our study identified six novel SNPs associated with differential risk of asthma exacerbations. In particular, we found that our top GWAS result, rs2395672, was present among the top‐ranked discovery SNPs and also replicated in PMRP (Tables 2 and 3). By meta‐analysis, the top two SNPs were associated with increased risk of exacerbations, while the remainder was associated with decreased risk (Table 3). Finally, we determined that the top candidate gene, *CMTR1*, was over‐expressed in nasal lavage samples from patients experiencing exacerbations in an independent microarray data set (Fig. 2), providing evidence for a role for this gene as a risk locus for exacerbations.

Among the six replicated SNPs, we identified SNPs present in three candidate genes that were not previously associated with asthma exacerbations (listed in Table 3). The top ranked gene, *CMTR1*, was tagged by intronic SNP rs2395672. *CMTR1* encodes hMTr1, a methyltransferase that catalyzes specific methylation of the 2′‐O‐ribose of the first nucleotide (cap1) of capped RNA transcripts 39. mRNA capping enhances mRNA stability and promotes efficient mRNA translation 40, 41. In addition, it serves as part of the innate host defense mechanism, as uncapped RNAs including viral transcripts, trigger an interferon‐mediated, antiviral response 42. hMTr1 is an interferon‐stimulated protein that participates in cellular defense mechanisms against viral infection 43. Up‐regulated *CMTR1* expression, along with other interferon stimulated genes, was associated with T‐cell mediated immune responses in human peripheral blood mononuclear cells (PBMC) 44. Thus, by regulating mRNA stability, and transcriptional expression of interferon‐induced genes, *CMTR1* may play an important role in regulating genes involved in immune responses to viral infections. Respiratory viruses are major triggers of exacerbations, and major cause of morbidity and mortality in adults and children with asthma 45. We found that rs2395672 in *CMTR1* is associated with an increased risk of exacerbations in genome‐wide genotype data from two independent asthmatic populations (Table 3). Furthermore, *CMTR1* mRNA transcripts were significantly up‐regulated in nasal lavage samples, which included a predominance of immune cell populations, from asthmatic children during a picornavirus‐induced exacerbation 35 (Fig. 2A). Consistent with the current knowledge of the biological functions of *CMTR1*, these data point toward a mechanistic role for this gene in the pathogenesis of asthma exacerbations.

In addition to *CMTR1,* we also identified *TRIM24* and *MAGI2* as associated with exacerbations. The SNPs in these genes were also present in introns. No roles for *TRIM24* have been described for asthma, respiratory disease, respiratory immune responses or corticosteroid use. We identified rs2691529 in *MAGI2* that was associated with reduced risk of exacerbations, but did not find that *MAGI2* was significantly differentially expressed in nasal lavage samples during or post exacerbation. However, a recent study showed that *MAGI2* was associated with airway wall thickening and bronchial inflammation, and increased *MAGI2* expression was also associated with fewer inflammatory cells in bronchial biopsies of patients with COPD 46. *MAGI2* was also reportedly associated with allergy in a GWAS of allergic diseases in a Russian population 47. To gain insight into the functional roles for the SNP associations listed in Table 3, we explored whether they are predicted to alter the expression of other genes and serve as expression quantitative trait loci (eQTLs) using the SNP and Copy Number Variant (CNV) Database (SCAN; http://www.scandb.org/). While rs2395672 was not predicted to be an eQTL, three of the SNPs listed in Table 3 could predict expression for multiple genes (*P* < 0.0001; CEU population): rs279728 (*AMT*; *NICN1*), rs2691529 (*CCNJ*) and rs9303988 (*IMPA2*; *LOC100128510*; *ZNF75A*). However, the roles of these genes in asthma are unknown. Further genetic and molecular studies are needed to elucidate the function of these SNPs and genes in asthma pathogenesis.

Our study has multiple strengths, with potential benefits for asthma therapy. First, we identified genetic risk factors associated with persistent exacerbations in patients taking ICS, which represents a severe, understudied asthmatic phenotype related to therapeutic outcomes. Using genetic information to help identify patients who are more likely to experience persisting asthma exacerbations, despite treatment with ICS, has significant value. Using this information, clinicians can consider alternative controller medications, and pursue additional asthma management interventions, such as increased outpatient follow‐up visits for patients who are more likely to experience exacerbations while taking ICS. Secondly, we evaluated samples and patient medical records from well‐phenotyped populations from the Vanderbilt and Marshfield biobank resources. Studying a population of patients, rather than subjects in a clinical trial, increases the generalizability of our findings to other asthmatic populations. Furthermore, we demonstrate the applicability of mining EMR data for patient selection, development of clinical outcome measures, and for guiding large‐scale genomic studies. In addition, our sample size of 806 patients is one of the largest used to evaluate the pharmacogenetics of ICS response. We anticipate that these results will inform future meta‐analyses and serve as replication for studies with larger cohorts.

Despite the strengths of our study, several limitations should be mentioned. Foremost, none of our SNPs achieved genome‐wide significance. Second, the effect size estimates for these associations are small, although this is a common result for many complex trait GWA studies. Third, we only evaluated Caucasians, limiting generalizability of our study to a diverse population, but this may also be considered a strength as we were able to limit population stratification and thereby improve the interpretability of our findings. Fourth, inclusion of additional demographic data (e.g., disease severity, atopy, smoking status) could also help to determine whether the genetic associations with exacerbations are independent of these conventional risk factors for asthma exacerbations. Finally, the expression data were derived from an independent data set and not directly from the DNA of the biobank patients, although this could also serve as an independent validation of the mechanistic relevance of *CMTR1*.

In conclusion, genetic variability appears to contribute to asthma exacerbations in patients taking ICS. Our studies implicate several new loci, including *CMTR1*, a novel candidate gene with a potential role in the pathogenesis of asthma exacerbations.
